# Respiratory Metabolism Responses of Chinese Mitten Crab, *Eriocheir sinensis* and Chinese Grass Shrimp, *Palaemonetes sinensis*, Subjected to Environmental Hypoxia Stress

**DOI:** 10.3389/fphys.2018.01559

**Published:** 2018-11-06

**Authors:** Jie Bao, Xiaodong Li, Han Yu, Hongbo Jiang

**Affiliations:** ^1^Liaoning Provincial Key Laboratory of Zoonosis, College of Animal Science and Veterinary Medicine, Shenyang Agricultural University, Shenyang, China; ^2^Research and Development Center, Panjin Guanghe Crab Industry Co., Ltd., Panjin, China

**Keywords:** *Eriocheir sinensis*, *Palaemonetes sinensis*, environmental hypoxia, oxygen consumption rate, ammonia excretion rate, O:N ratio, succinate dehydrogenase, lactate dehydrogenase

## Abstract

Environmental hypoxia represents a major physiological challenge for *Eriocheir sinensis* and *Palaemonetes sinensis* and is a severe problem in aquaculture. Therefore, understanding the metabolic response mechanisms of *E. sinensis* and *P. sinensis*, which are economically important species, to environmental hypoxia and reoxygenation is essential. However, little is known about the intrinsic mechanisms by which *E. sinensis* and *P. sinensis* cope with environmental hypoxia at the metabolic level. Hypoxia–reoxygenation represents an important physiological challenge for their culture. In this study, respiratory metabolism and respiratory metabolic enzymes of *E. sinensis* and *P. sinensis* were evaluated after different hypoxia and reoxygenation times. The results showed that environmental hypoxia had a dramatic influence on the respiratory metabolism and activities of related enzymes. The oxygen consumption rates (OCR) significantly increased as hypoxia time increased, while the ammonia excretion rate (AER) was significantly lower than that in the control group after 8 h hypoxia. The oxygen to nitrogen ratio (O:N) in the control group was <16, indicating that all the energy substrates were proteins. After environmental hypoxia, the O:N significantly increased, and the energy substrate shifted from protein to a protein–lipid mixture. The OCR, AER, and O:N did not restore to initial levels after 2 h or 12 h reoxygenation and was still the same as after 8 h hypoxia. As environmental hypoxia time increased, succinate dehydrogenase (SDH) gradually decreased and lactate dehydrogenase (LDH) gradually increased. Both SDH and LDH were gradually restored to normal levels after reoxygenation. Therefore, environmental hypoxia should be avoided as much as possible during aquaculture breeding of *E. sinensis* and *P. sinensis*. Further, since OCR will significantly increase after a short period of reoxygenation, secondary environmental hypoxia due to rapid consumption of oxygen should also be avoided in aquaculture.

## Introduction

The growth and development of crustaceans is regulated by the endocrine system, and aspects of the external environment, such as dissolved oxygen (DO), temperature, salinity, light, and pH (Castejón et al., 2015; Long et al., 2017). The level of DO is a key indicator of water quality, and partially determines the intensity of crustacean aquaculture. A suitable level of DO enables the survival of crustaceans. There are many factors that cause hypoxia in aquaculture, such as extreme weather changes, eutrophication, long-distance transportation, and human factors (Lehtonen and Burnett, 2016; Lund et al., 2017). Much research has focused on the negative effects of environmental hypoxia on crustaceans. For example, environmental hypoxia causes abnormal breathing and metabolic disorders in crustaceans, which leads to a decline in feeding and efficiency of food transformation and retarded growth (Han et al., 2017; Sun et al., 2018). Environmental hypoxia even affects the behavior, morphological characteristics, immunity, and reproduction of crustaceans (De Lima et al., 2015; Lara et al., 2017; Peruzza et al., 2018.).

The Chinese mitten crab *Eriocheir sinensis* is an important freshwater crustacean that is mainly cultured in the Yangtze, Yellow, and Liaohe River systems. In 2015, the total production of *E. sinensis* was about 823,259 t, accounting for 30% of the production of crustaceans in freshwater aquaculture (Bureau of Fisheries, Management of Chinese Ministry of Agriculture, 2016). Chinese grass shrimp *Palaemonetes sinensis* is the only *Palaemonetes* species recorded in China. This shrimp is mainly distributed in northern China and Siberia in Russia. *P. sinensis* not only has high ornamental and consumer value, but also has important ecological value in lakes, rivers, and reservoirs (Dong et al., 2018). In recent years, with the depletion of natural resources, artificial culture of *P. sinensis* has been rapidly developed. Farming models continues to innovate, and at present *P. sinensis* is one of the most important species cultivated in rice fields in China (Li et al., 2018).

*Eriocheir sinensis* and *P. sinensis* use an ex-gill to exchange DO in the water and CO_2_ in the hemolymph to complete respiration. Under artificial breeding conditions, excessive feeding can easily cause excessive accumulation of organic matter on the bottom of culture tanks, along with excreta. The increasing density of cultures exacerbates the lack of DO, especially at night and early in the morning. At present, the solution is relatively simple, and mainly relies on an open aerator to maintain a high oxygen content in the water. However, these methods cannot solve the problem of oxygen deficiency completely. As the breeding of crustaceans intensifies, the problem of environmental hypoxia becomes increasingly prominent.

In hypoxic conditions, tissues must either increase anaerobic energy production, improve energy use, or lower energy consumption. Respiratory metabolism is the basic physiological activity of energy metabolism in living organisms, and not only reflects metabolic characteristics, physiological state, and nutritional status of the organism, but also the effects of environmental conditions on biological physiological activities (Leiva et al., 2015). Respiration is one of the important components of physiological studies of aquatic animals. Oxygen consumption rate (OCR), ammonia excretion rate (AER), and oxygen to nitrogen ratio (O:N) are important indicators of the respiratory metabolism of aquatic animals, and can be used to assess the energy use patterns of aquatic animals and their tolerance to environmental hypoxia (Zhang et al., 2014). During metabolic processes, specific enzymes accelerate the conversion of metabolites to produce energy to meet the needs of the body, and the changes in the activity of the respiratory metabolic enzymes are a direct reflection of the changes in metabolic capacity. Succinate dehydrogenase (SDH) is involved in the tricarboxylic acid cycle and oxidative phosphorylation, and its activity partially reflect levels of aerobic metabolism (Rutter et al., 2010). Lactate dehydrogenase (LDH) catalyzes the conversion of pyruvate to lactic acid, which is a marker enzyme of anaerobic metabolism, and regarded as a stress indicator (Albert and Ellington, 1985). Therefore, SDH and LDH activities reflect the levels of aerobic and anaerobic metabolism, respectively.

This study used *E. sinensis* and *P. sinensis* to study the changes in respiratory metabolism and respiratory metabolic enzyme activity (SDH and LDH) after environmental hypoxia stress. The results provide a theoretical basis for the development of freshwater aquaculture of *E. sinensis* and *P. sinensis*.

## Materials and Methods

### Ethics Statement

All animals were handled in accordance with the permits which were established by Animal Experiments Ethics Committee of Shenyang Agricultural University for the care and use of laboratory animals.

### Experimental Animals

*Eriocheir sinensis* and *P. sinensis* were obtained from Panjin Guanghe Crab Industry Co., Ltd. in Panjin, Liaoning Province, China. Individuals were transported to Shenyang Agricultural University (Liaoning Province) in insulated polystyrene foam boxes containing water-soaked cloths and frozen gel. At the laboratory, *E. sinensis* and *P. sinensis* were distributed between four 300-L aquaria (100 crabs and 200 shrimps per aquarium). To ensure the oxygen content was maintained above 7 mg⋅L^-1^, constant aeration was provided by air pumps in each aquarium. The pH was 7.0–7.2 and a natural photoperiod of 12:12 h (light:dark) was used. *E. sinensis* and *P. sinensis* were fed artificial feed twice per day. Leftovers were removed 2 h after feeding. All the water was renewed every day. After a 2-week acclimation period, healthy and energetic *E. sinensis* and *P. sinensis* were used in the experiments.

### Experimental Methods

#### Experimental Design

**FIGURE 1:**
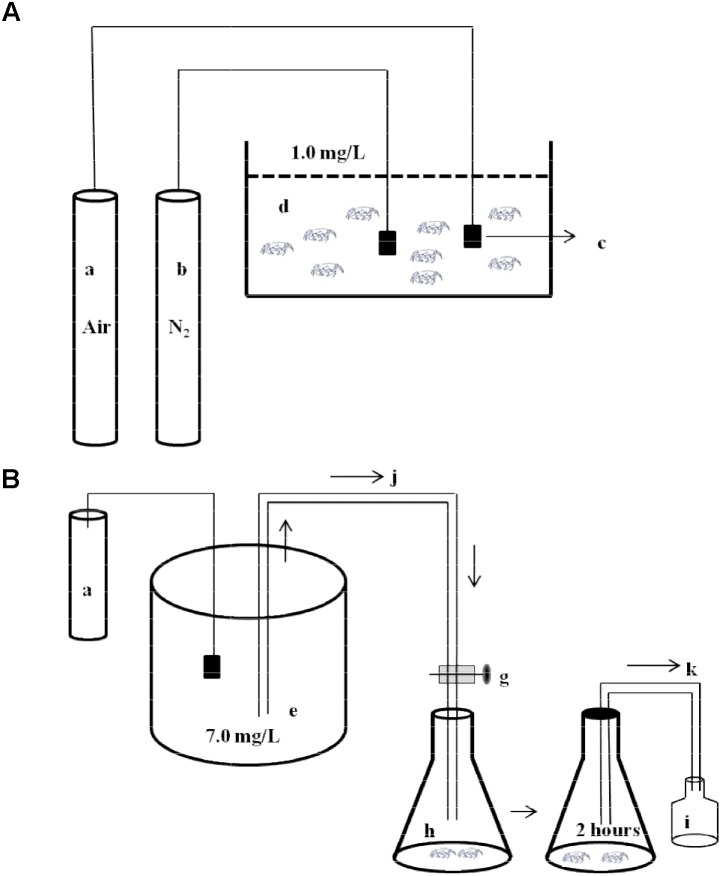
Schematic diagram of experimental design. **(A)** The diagram of environmental hypoxia stress to experimental animals. A part of stress animals were dissected to analyze activities of succinate dehydrogenase (SDH) and lactate dehydrogenase (LDH). Another part stress animals were transfer to the respiratory chambers to measure oxygen consumption rate (OCR) and ammonia excretion rate (AER). a, air pump; b, nitrogen container; c, air stone; d, hypoxia aquarium (DO 1.0 ± 0.2 mg⋅L^-1^). **(B)** The diagram of the respirometer. e, water reservoir (DO 7.0 ± 0.2 mg⋅L^-1^); g, valves; h, respiratory chamber; i, sample bottle; j, inflow; k, outflow. (OCR and AER measurement: water (DO 7.0 ± 0.2 mg⋅L^-1^) gently flowed into the respiratory chamber and then were sealed immediately using transparent plastic film. The experiment was carried out for 2 h. Initial and final DO and ammonia-nitrogen concentration were measured.).

A schematic diagram of experimental design was shown in Figure 1. *E. sinensis* (mean body mass 3.66 ± 0.18 g) and *P. sinensis* (mean body mass 0.24 ± 0.03 g) with similar body weights were selected. Individuals were starved for 24 h before the experiment. In the control group, the air pump provided continuous aeration to maintain DO at 7.0 ± 0.2 mg⋅L^-1^. The experimental groups were aerated with nitrogen and air to maintain DO at 1.0 ± 0.2 mg⋅L^-1^. Hypoxia time lasted 8 h, then the DO was restored to 7.0 ± 0.2 mg⋅L^-1^, and reoxygenation time lasted 12 h. Samples were measured after 0, 2, 4, and 8 h hypoxia, and after 2 h (R2 h) and 12 h (R12 h) reoxygenation. During the experiment, the concentration of DO in the aquaria was monitored and adjusted using the ventilation volume over time. No individuals died during the experiment.

#### Oxygen Consumption Rate and Ammonia Excretion Rate

Crabs and shrimps were reared in separate aquaria (500 mm × 250 mm × 300 mm). Experimental animals were randomly chosen after 0, 2, 4, and 8 h environmental hypoxia, and after 2 and 12 h reoxygenation. The experiment was carried out in a 1-L-triangular cone bottle. Two crabs or six shrimps were put in each bottle. Four replicates were established for each time point and 4 blank aerated bottles (no animals) were used as controls. Water gently flowed into the bottles until it overflowed the rims continuously. The bottles were then sealed immediately using transparent plastic film. DO was 7.0 ± 0.2 mg⋅L^-1^. The experiment was carried out for 2 h. Initial and final DO and NH_4_-N were measured using the Winkler titration and the indophenol methods, respectively (Strickland and Parsons, 1972). At the end of the experiment, wet weight was measured immediately after blotting excess water with filter paper.

### Enzyme Assays

After 0, 2, 4, and 8 h environmental hypoxia and 2 and 12 h reoxygenation, crabs and shrimps were randomly sampled and frozen in liquid nitrogen to analyze enzyme activities. Four replicates were established for each time point. Sample of gills were taken from each individual. A glass homogenizer was used to homogenize the gills on ice for 2 min. The frozen samples were treated with 5 volumes of 0.05 M PBS buffer (pH 7.5). The homogenate was centrifuged for 8 min at 3500 ×*g*, and the supernatant was collected and kept at -80°C. All samples were tested using analysis kits manufactured by Nanjing Jiancheng Bioengineering Institute (Nanjing, Jiangsu province, China). The activities of LDH (EC 1.1.1.27) and SDH (EC 1.3.5.1) were measured according to the manufacturer’s instructions. SDH enzyme activity unit was defined as: the absorbance of the reaction system (including 0.1 mol/L sodium succinate, 0.01% methylene blue, 0.04 mol/L disodium malonate, and pH 7.0 phosphate buffers) was reduced by 0.01 per minute per milligram of tissue protein at 37°C. For the determination of LDH activity, an enzyme activity unit was defined as producing 1 μmol pyruvic acid in the reaction system (including coenzyme I, 4 mol/L NaOH, 2,4-dinitrophenylhydrazine, and 2 mmol/L sodium pyruvate) at 37° C for 15 min per gram of tissue protein. Total soluble protein concentrations used for calculation of enzyme-specific activities were determined according to Bradford (1976) using bovine serum albumin as the standard. SDH and LDH were expressed as U/mg prot.

### Data Calculation and Statistical Analysis

The standard metabolic rate was evaluated in terms of OCR, AER, and O:N.

$$\begin{array}{l} \text{OCR}(\text{mg}\cdot {\text{g}}^{-1}\cdot {\text{h}}^{-1})=\frac{\left(\text{DO}1-\text{DO}2\right)\times \text{V}}{\text{t}\times \text{W}}\\ \text{AER}(\text{mg}\cdot {\text{g}}^{-1}\cdot {\text{h}}^{-1})=\frac{\left(\text{N}2-\text{N}1\right)\times \text{V}}{\text{t}\times \text{W}}\\ \;\text{O}:\text{N}=\frac{14\times \text{OCR}}{16\times \text{AER}} \end{array}$$

where DO1 is the oxygen concentration at the beginning of the metabolic experiment (mg O_2_⋅L^-1^), DO2 is the oxygen concentration at the end of the metabolic experiment (mg O_2_⋅L^-1^), V is the volume of the respiration chamber (L), t is the duration of the metabolic experiment (h), W is the body weight of the animals (g, wet weight), N1 is the ammonia-nitrogen concentration in the water of the experimental chamber at the end of the metabolic experiment (mg NH_3_-N⋅L^-1^), and N2 is the ammonia-nitrogen concentration in the water of the experimental chamber at the beginning of the metabolic experiment (mg NH_3_-N⋅L^-1^). The O:N is an important indicator of animal metabolism, as it represents the ratio of fat, protein, and carbohydrate catabolism inside the organism. It is expressed as the ratio of oxygen to nitrogen atoms (Ahmed et al., 2008). Values are presented as mean ± SE. All indices were calculated and analyzed using SPSS 20.0 for windows. A one-way ANOVA was used to compare the differences in OCR, AER, O:N, LDH, and SDH activities, followed by Tukey’s multiple comparison tests. Comparing the data of the two different groups at each time point of OCR, AER, O:N, LDH, and SDH activities, the Student’s *t*-test with two-tails was employed. Differences were considered significant at *P* < 0.05.

## Results

### Effects of Environmental Hypoxia on Respiratory Metabolism of *E. sinensis* and *P. sinensis*

#### Oxygen Consumption Rate

**FIGURE 2:**
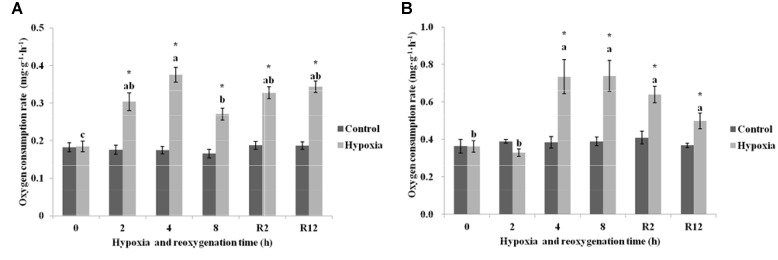
Effect of environmental hypoxia on OCR of *Eriocheir sinensis* **(A)** and *Palaemonetes sinensis* **(B)**. There was no significant difference between bars with the same letter (*P* < 0.05). Asterisk represents a significant difference between the control and hypoxia groups at the same time point (*P* < 0.05).

As shown in Figure 2A, the OCR in the control group of *E. sinensis* was not significantly different over time. However, the OCR increased first and then decreased as environmental hypoxia time increased, and there was a significant difference between the hypoxia and control groups (*P* < 0.05). After reoxygenation, the OCR was not significantly difference from that at 2, 4, or 8 h hypoxia (*P* > 0.05), and was still significantly higher than that in the control group (*P* < 0.05).

Figure 2B showed that the OCR in the control group of *P. sinensis* was also not significantly different over time. The OCR after 4 and 8 h hypoxia was significantly higher than that in the control group (*P* < 0.05), but there was no difference between OCR after 2 h hypoxia and that in the control group (*P* > 0.05). After DO returned to normal, the OCR gradually decreased, but was still significantly different from that in R2 h, R12 h, and the control groups (*P* < 0.05).

#### Ammonia Excretion Rate

**FIGURE 3:**
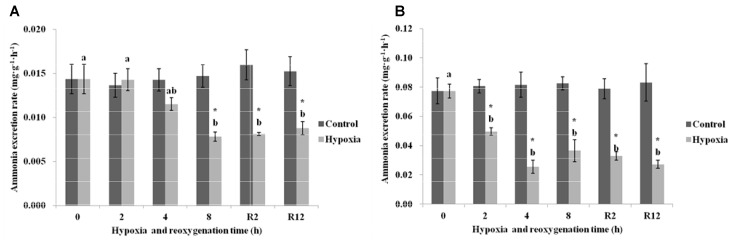
Effect of environmental hypoxia on AER of *E. sinensis* **(A)** and *P. sinensis* **(B)**. There was no significant difference between bars with the same letter (*P* < 0.05). Asterisk represents a significant difference between control and hypoxia groups at the same time point (*P* < 0.05).

As shown in Figure 3A, the AER in the control group of *E. sinensis* was not significantly different over time. In the hypoxia group, the AER began to gradually decrease, and decreased significantly after 8 h hypoxia (*P* < 0.05). The AER after R2 h and R12 h reoxygenation was significantly lower than that in the control group and after 2 h hypoxia (*P* < 0.05), but not significantly different from that after 4 and 8 h hypoxia (*P* > 0.05).

Figure 3B showed that the AER in the control group of *P. sinensis* was also not significantly different over time. The AER in the hypoxia group was significantly lower than that in the control group (*P* < 0.05), even after returning to normal DO levels, the AER was still significantly lower than that of the control group (*P* < 0.05).

#### O:N

**FIGURE 4:**
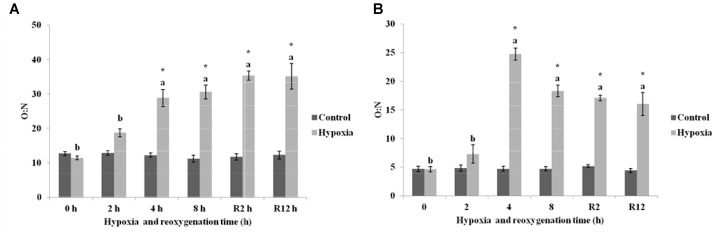
Effect of environmental hypoxia on oxygen to nitrogen ratio of *E. sinensis* **(A)** and *P. sinensis* **(B)**. There was no significant difference between bars with the same letter (*P* < 0.05). Asterisk represents a significant difference between control and hypoxia groups at the same time point (*P* < 0.05).

Figure 4A showed that the O:N in the control group of *E. sinensis* was not significantly different over time. The O:N began to increase gradually as environmental hypoxia time increased and was significantly higher than that in the control group after 4 and 8 h hypoxia (*P* < 0.05). After DO returned to normal, the O:N was still significantly higher than that in the control group (*P* < 0.05), and not significantly different from that after 4 and 8 h hypoxia (*P* > 0.05).

As shown in Figure 4B, the O:N in the control group of *P. sinensis* was also not significantly different over time. The O:N in *P. sinensis* showed the same variation as *E. sinensis* and gradually increased as environmental hypoxia time increased. The O:N after 4 and 8 h hypoxia was significantly higher than that in the control group (*P* < 0.05). After DO returned to normal, the O:N was still significantly higher than that in the control group (*P* < 0.05).

### Effect of Environmental Hypoxia on Respiratory Metabolic Enzymes of *E. sinensis* and *P. sinensis*

#### SDH

**FIGURE 5:**
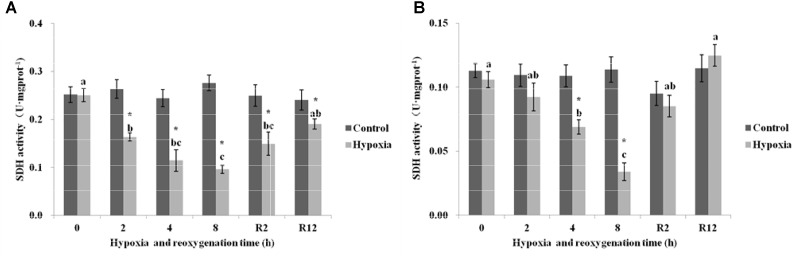
Effect of environmental hypoxia on SDH of *E. sinensis* **(A)** and *P. sinensis* **(B)**. There was no significant difference between bars with the same letter (*P* < 0.05). Asterisk represents a significant difference between control and hypoxia groups at the same time point (*P* < 0.05).

As shown in Figure 5A, the SDH in the control group of *E. sinensis* was not significantly different over time. The SDH began to gradually decrease as environmental hypoxia time increased. Compared with the control group, SDH in the hypoxia group significantly decreased (*P* < 0.05). The lowest SDH activity was 0.096 U⋅mg prot^-1^ at 8 h. After DO returned to normal, the SDH gradually increased and recovered to normal levels at R12 h and was not significantly different from that in the control group (*P* > 0.05). However, the SDH at R2 h was still significantly higher than that in the control group (*P* < 0.05).

Figure 5B showed that the SDH in the control group of *P. sinensis* was also not significantly different over time. However, in the hypoxia group, the SDH decreased gradually as environmental hypoxia time increased, and was significantly different from that in the control group after 4 and 8 h hypoxia (*P* < 0.05). The lowest SDH activity was 0.034 U⋅mg prot^-1^ at 8 h. After DO returned to normal, the SDH gradually increased and recovered to normal levels at R2 h and R12 h, and there was no significant difference from that in the control group (*P* > 0.05).

#### LDH

**FIGURE 6:**
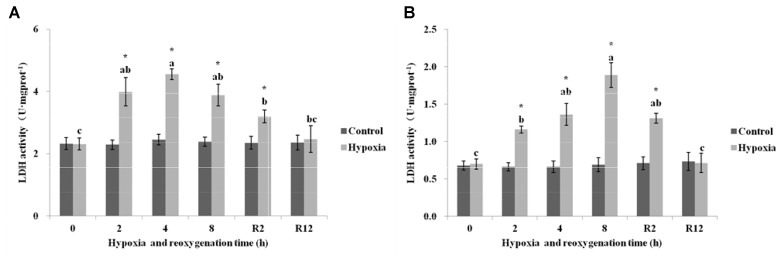
Effect of environmental hypoxia on LDH of *E. sinensis* **(A)** and *P. sinensis* **(B)**. There was no significant difference between bars with the same letter (*P* < 0.05). Asterisk represents a significant difference between control and hypoxia groups at the same time point (*P* < 0.05).

As shown in Figure 6A, the LDH in the control group of *E. sinensis* was not significantly different over time. However, the LDH significantly increased after 2, 4, and 8 h hypoxia (*P* < 0.05). The highest LDH activity was 4.55 U⋅mg prot^-1^ at 4 h. After DO returned to normal, the LDH gradually decreased and recovered to normal levels at R12 h and was not significantly different from that in the control group (*P* > 0.05).

Figure 6B showed that the LDH in the control group of *P. sinensis* was also not significantly different over time. The LDH began to increase gradually as environmental hypoxia time increased. There was a significant difference between LDH after 2, 4, and 8 h hypoxia and that in the control group (*P* < 0.05). The highest LDH activity was 1.89 U⋅mg prot^-1^ at 8 h hypoxia. After DO returned to normal, the LDH decreased gradually and recovered to normal levels at R12 h and was not significantly different from that in the control group (*P* > 0.05). The LDH at R2 h was still higher than that in the control group (*P* < 0.05).

## Discussion

Oxygen consumption rates and AER are important indicators of the metabolic activity of aquatic organisms. Studies have shown that circadian rhythm, size, amount of food, temperature, and salinity can all influence the OCR and AER of organisms (Crear and Forteath, 2000). In this study, the OCR of *E. sinensis* and *P. sinensis* increased as environmental hypoxia time increased. This may explain why oxygen demand rapidly increased to compensate for the lack of oxygen supply in the body after extended hypoxia. After 12 h reoxygenation, the OCR of *E. sinensis* and *P. sinensis* was still significantly higher than that in the control group, indicating that it was difficult to recover to normal levels in such a short period of time. DO in ponds varies markedly between day and night, which may impose significant effects on *E. sinensis* and *P. sinensis*, particularly in rearing ponds without aerators during the heat of summer. *E. sinensis* and *P. sinensis* always suffered environmental hypoxia at night due to their respiration and the decomposition of accumulated organic matter from unconsumed food and feces (Cheng et al., 2003). Oxygen content gradually increases after sunrise; however, from our results, it is evident that *E. sinensis* and *P. sinensis* will consume more oxygen during the reoxygenation stage, and that OCR will rapidly increase. If oxygen supply is insufficient, it will be exhausted quickly, presenting a risk of continuing environmental hypoxia.

The nitrogen excretion products of crustaceans mainly include ammonia, uric acid, urea, and amino acids. It is generally thought that exogenous nitrogen excretion and deamination produce ammonia nitrogen, and that endogenous nitrogen protein and nucleotides decompose to produce urea. Amino acids are the main sources of ammonia production *in vivo*. Crustaceans can regulate the concentration of intracellular free amino acids to cope with environmental stress (Greenaway, 1991). In this study, the AER of *P. sinensis* after environmental hypoxia was significantly lower than that in the control group, while the AER of *E. sinensis* decreased gradually as environmental hypoxia time increased, and was also significantly lower than that in the control group at 8 h. After 12 h reoxygenation, the AER of R2 h and R12 h did not return to normal levels, and was still significantly lower than that in the control group. These results showed that the metabolic patterns of *E. sinensis* and *P. sinensis* changed in response to environmental hypoxia stress, while short-term reoxygenation did not enable recovery to the initial nitrogen excretion levels.

Oxygen to nitrogen ratio has been widely used to determine the nature of oxidized metabolic substrates in aquatic animals under stress conditions (Dall and Smith, 1986). Theoretically, catabolism of pure protein produces an O:N from 3 to 16, and equal proportions of proteins and lipid results in an O:N from 50 to 60. Values >60 correspond to lipids and carbohydrates (Mayzaud and Conover, 1988). Intriguingly, in crustaceans, the preferred metabolic substrate appears to differ with salinity. In general, freshwater crustaceans rely mainly on carbohydrate metabolism, while marine species tend toward protein metabolism (Clifford and Brick, 1983). In this study, the metabolic substrates of *E. sinensis* and *P. sinensis* in the control group were proteins and were more similar to seawater crustaceans. This may be related to their individual biological characteristics. *E. sinensis* is a migratory crab that must reproduce in seawater, while *P. sinensis* does not need to migrate to reproduce, but can survive for a long time in 28 ppt seawater (data unpublished). Normally, crustaceans will change their metabolite substrates to adapt to environmental change. For example, *Litopenaeus stylirostris* maintained at 6 mg⋅L^-1^ use carbohydrates as their main energy substrate, but when exposed to an oxygen concentration of 4 mg⋅L^-1^, their main energy substrate was a protein–lipid mixture, and at 2 mg⋅L^-1^, protein was the main energy substrate during stress response (Re and Díaz, 2011). In this study, the O:N value increased from 11.46 to 28.83 at 4 h environmental hypoxia and to 30.59 at 8 h environmental hypoxia in *E. sinensis*. In *P. sinensis*, it increased from 7.24 to 24.75 at 4 h environmental hypoxia and to 18.31 at 8 h environmental hypoxia, which demonstrated a shift in nutrient utilization from proteins to protein–lipid mixture. These results are similar to those reported by Chandurvelan et al. (2017) for *Paratya curvirostris*. Cadmium induced an increased in O:N, which was explained by an increased reliance on carbohydrates and/or lipids as metabolic substrates, which were stimulated by the increased metabolic costs of environmental hypoxia. We also found that glucose level significantly increased after environmental hypoxia (data unpublished). Unexpectedly, the metabolic substrate was still a protein–lipid mixture after 12 h reoxygenation. It may be that long-term environmental hypoxia leads to excessive energy consumption, and that metabolic level cannot be restored to the initial level after such a short period of reoxygenation. Therefore, when breeding crustaceans, it may benefit healthy growth of crustaceans to adjust the proportion of nutrients in the feed, according to the oxygen conditions at different feeding times.

Succinate dehydrogenase and LDH are very important enzymes in respiratory metabolism. SDH is a connection oxidative phosphorylation and an electron transfer center and is the only enzyme in the TCA cycle mosaic in the mitochondrial membrane. As a symbol of mitochondrial enzyme activity, size directly affects the rate of the TCA cycle. In general, SDH activity is used as an important indicator to measure the aerobic capacity of the body (Simon and Robin, 1971). These results showed that significantly lower SDH activity occurred in *E. sinensis* and *P. sinensis* after environmental hypoxia, indicating a lower level of aerobic metabolism, similar to a previous study of *Macrobrachium nipponense* (Sun et al., 2018). LDH is a key regulatory enzyme in anaerobic glycolysis. It exists in the cytoplasm and can control and catalyze the transformation from pyruvate to lactic acid. Therefore, LDH activity is often used as an important indicator of anaerobic metabolic capacity (Viru, 1994). In this study, the content of SDH gradually decreased and LDH content gradually increased as environmental hypoxia time increased. The aerobic capacity of *E. sinensis* and *P. sinensis* decreased and anaerobic respiration ability began to rise after environmental hypoxia. These findings suggest that environmental hypoxia results in a shift from aerobic to anaerobic metabolism, and that sufficient ATP can be generated only by upregulating anaerobic metabolism in hypoxic crustaceans. Other studies have also shown that the hypoxic environment at high altitude can lead to decreased SDH activity and increased LDH activity in Qinghai lizards (He et al., 2013). Valarmathi and Azariah (2003) exposed the estuarine crab *Sesarma quadratum* to copper and found that the activity of LDH increased and SDH decreased in the muscle, gill, and hepatopancreas. After 12 h reoxygenation, the content of SDH gradually increased and the content of LDH gradually decreased, which restored them to the level of the control group. These results showed that the activity of SDH and LDH rapidly recovered to normal levels after reoxygenation.

## Conclusion

This study showed that environmental hypoxia had a dramatic influence on the respiratory metabolism and activities of related enzymes of *E. sinensis* and *P. sinensis*. The OCR of *E. sinensis* and *P. sinensis* increased as environmental hypoxia time increased. However, the AER of *P. sinensis* after hypoxia was significantly lower than that in the control group. The energy substrate shifted from protein to a protein–lipid mixture. As environmental hypoxia time increased, the content of SDH gradually decreased while LDH content gradually increased. Therefore, when breeding crustaceans, environmental hypoxia should be avoided as much as possible. If hypoxia occurs, OCR will significantly increase after reoxygenation, and secondary environmental hypoxia due to rapid consumption of oxygen should also be avoided.

## Author Contributions

JB and HJ designed the research and wrote the manuscript. HY and JB performed the majority of the experiments, data processing, analysis, and interpretation. XL assisted in sample collection and detection of indicators. HJ and XL revised the manuscript.

## Conflict of Interest Statement

XL was employed by Panjin Guanghe Crab Industry Co., Ltd., Panjin, Liaoning, China.

The remaining authors declare that the research was conducted in the absence of any commercial or financial relationships that could be construed as a potential conflict of interest.
